# MicroRNA—A Tumor Trojan Horse for Tumor-Associated Macrophages

**DOI:** 10.3390/cells8121482

**Published:** 2019-11-21

**Authors:** Shahzad Nawaz Syed, Ann-Christin Frank, Rebecca Raue, Bernhard Brüne

**Affiliations:** 1 Institute of Biochemistry I, Faculty of Medicine, Goethe-University Frankfurt, 60590 Frankfurt, Germany; syed@biochem.uni-frankfurt.de (S.N.S.); frank@biochem.uni-frankfurt.de (A.-C.F.); raue@biochem.uni-frankfurt.de (R.R.); 2 Project Group Translational Medicine and Pharmacology TMP, Fraunhofer Institute for Molecular Biology and Applied Ecology, 60596 Frankfurt, Germany; 3 German Cancer Consortium (DKTK), Partner Site Frankfurt, 60590 Frankfurt, Germany; 4 Frankfurt Cancer Institute, Goethe-University Frankfurt, 60596 Frankfurt, Germany

**Keywords:** microRNA, tumor-associated macrophages, macrophage polarization, CD36, breast cancer, inflammation, miR-375, exosomes, LDL

## Abstract

MicroRNAs (miRs) significantly contribute to the regulation of gene expression, by virtue of their ability to interact with a broad, yet specific set of target genes. MiRs are produced and released by almost every cell type and play an important role in horizontal gene regulation in the tumor microenvironment (TME). In the TME, both tumor and stroma cells cross-communicate via diverse factors including miRs, which are taking central stage as a therapeutic target of anti-tumor therapy. One of the immune escape strategies adopted by tumor cells is to release miRs as a Trojan horse to hijack circulating or tumor-localized monocytes/macrophages to tune them for pro-tumoral functions. On the other hand, macrophage-derived miRs exert anti-tumor functions. The transfer of miRs from host to recipient cells depends on the supramolecular structure and composition of miR carriers, which determine the distinct uptake mechanism by recipient cells. In this review, we provide a recent update on the miR-mediated crosstalk between tumor cells and macrophages and their mode of uptake in the TME.

## 1. Introduction

Cellular and molecular communications are the basis of ontogeny and oncogeny. Every living being relies on communication in order to survive and propagate. Pathophysiological situations such as cancer are no exception and rely on this indispensable tool for initiation and propagation. There are various ways in which cancer and host cells communicate with each other, including soluble factors, cell‒cell contact, or complex macromolecule interactions, i.e., receptor-mediated events. This ultimately culminates in altered gene expression in participating cells [1,2,3,4]. Tumors comprise a heterogeneous cell composition. There are not only genetically distinct tumor cells, as a result of accumulating mutations, but also several immune cell types, including tumor-associated macrophages (TAMs), which play an important role in tumor development, invasion, and metastasis [2]. The phenotypic plasticity of macrophages includes an overlapping spectrum of dynamic responses, from classically activated (also known as M1) to alternatively activated (also known as M2). Classically activated macrophages are stimulated by lipopolysaccharide (LPS), interferon gamma (IFN-γ), and tumor necrosis factor (TNF-α), and provoke the secretion of pro-inflammatory cytokines including interleukin-1 (IL-1), IL-6, IL-12, IL-23, and TNF-α and reactive nitrogen and oxygen intermediates (RNI, ROI) [5]. In contrast, anti-inflammatory stimuli such as IL-4, IL-13, IL-10, and glucocorticoid or immune complexes (IC) plus LPS induce macrophages to an M2 phenotype. This type is characterized by a decreased production of pro-inflammatory cytokines, increased production of anti-inflammatory cytokines (e.g., IL-10), and factors that mediate immunosuppression and tissue remodeling. M2 macrophages have been divided into further subgroups. The M2a type is generated in response to IL-3 and IL-13, while M2b macrophages respond to immune complexes and Toll-like receptor (TLR) activation. M2c macrophages represent deactivated macrophages that suppress pro-inflammatory cytokines, while the M2d type represents a regulatory macrophage [6] that is often grouped with TAMs [5,7,8]. In line with these varied responses, Janus-faced macrophage functions are largely determined by their microenvironment rather than their genetic imprint [9,10]. In tumors and during the resolution of inflammation, hijacked macrophages attain pro-tumor or wound healing phenotypes due to tumor-derived factors [11], which emerge as a target for tumor therapy [12,13]. TAMs expressing classical activation markers produce pro-inflammatory cytokines and reactive oxygen species (ROS) and, thus, are crucial for tumor cell killing [14,15]. The high degree of plasticity demonstrated by macrophages in pathological conditions can be attributed to a) dynamic regulation of gene expression and b) rapid activation of signaling cascades that determine their effector functions. Signaling cascades triggered by local environmental cues and controlling the transcriptional machinery, ultimately determine the effector functions of macrophages in the tumor microenvironment (TME). Hence, understanding and modulating these aspects of macrophage pathobiology may have tremendous therapeutic options.

MicroRNAs (miRs) are small non-coding RNAs of about 22 nt, produced by almost all cells [16,17], that regulate gene expression at the post-transcriptional level [18]. MiR precursors are transcribed as long primary miRs (pre-miRs) by RNA polymerase II and subsequently processed by the microprocessor complex that contains the endoribonuclease DROSHA and its RNA binding partner DiGeorge Syndrome critical region gene 8 (DGCR8) or several less well-understood auxiliary factors such as the DEAD box RNA helicases p68 (also known as DDX5) and p72 (also known as DDX17) [19]. The resultant pre-miRs are exported to the cytoplasm via exportin 5, where they are further processed by the type III endoribonuclease DICER with the aid of the RNA-binding protein TAR. The RNA-binding protein (TRBP) and the interferon-inducible double-stranded RNA-dependent protein kinase activator A (PACT) produce double-stranded miR duplexes [20]. One of the strands is likely degraded, while mature miRs are loaded into the RNA-induced silencing complex (RISC). This complex consists of the Argonaute-2 (AGO2) protein and chaperones like HSC70/HSP90, which mediate the interaction of the guide strand of the miRNA with its target 3′-untranslated region (3′-UTR) of mRNAs, thereby repressing target gene expression [18,21,22,23,24]. Interestingly, there are also examples of miRs that induce transcription and upregulation of protein expression [25,26]. Nevertheless, the expression of pre-miRs is tightly regulated within cells at both the transcriptional and post-transcriptional level, which determines tissue-specific or ubiquitous expression profiles of mature miRs [27]. Mature miRs have the ability to bind to hundreds of mRNA targets, which themselves are also under tight and dynamic regulation [28]. Impaired miR expression is associated with a number of diseases, including cancer [29]. Calin et al. published a pioneering study that described how miRs and cancer are linked and identified that miR-15 and miR-16 were downregulated in most patients with chronic lymphocytic leukemia [30]. A growing body of literature has established that miRs are frequently deregulated in many types of cancer and are involved in tumor initiation and progression, metastasis, and chemotherapy resistance, pointing to the existence of oncogenic and tumor-suppressive miRs [31,32,33,34,35].

Phenotypic changes and the polarization of macrophages are also affected by miRs, which is critical to their involvement in tumor immunity [36]. Furthermore, miR-mediated gene regulation provides an extra layer of gene expression control. This allows an endogenous molecular switch of macrophage plasticity and effector functions in a pathological environment such as the TME. This review is a compendium of conundrums created by tumor-derived miRs and their impact on monocytes and macrophages for pro-tumor functions, with a special emphasis on the mechanisms of miR uptake by recipient cells.

## 2. Tumor-Derived miRs—Packaging Matters!

Tumor-derived factors such as cytokines, chemokines, growth factors, and small molecules have been shown to facilitate cellular communication between tumor cells and stroma [11,37,38,39]. Recent findings unravel another mode of communication, which is ascribed to the release of miRs into the extracellular fluid. Those extracellular miRs can act in a hormone-like fashion, in an autocrine or paracrine manner. Furthermore, tumor-derived miRs can act as a Trojan horse by hijacking stroma cells, particularly macrophages, to perform pro-tumoral functions [40]. Our recent findings suggest that the inherent anti-tumor activity of monocytes/macrophages can be reprogrammed by tumor-derived miR-375 at the initial phase of tumorigenesis by targeting these cells in the circulation, thereby attracting them to the TME for pro-tumoral functions [38]. This phenomenon is not surprising as the literature survey suggested that circulating miRs are considered as biomarkers. For instance, elevated levels of miR-155, miR-21, and miR-210 have been detected extracellularly in the sera of patients with diffuse large B-cell lymphoma [41]. The pioneering study of Valadi et al. demonstrated that extracellular vesicles containing mRNA and miR populations are released by mouse and human mast cells and can be taken up by recipient cells to alter gene expression [39]. As anticipated, the role of extracellular miRs in the cell‒cell communication of tumor and immune cells within the TME was documented as well [42,43].

There are several ways and molecular compositions in which tumors shed their miR repertoire, such as encapsulated in extracellular vesicles (EVs), or as nascent protein/lipoprotein‒miR complexes. The composition and final packaging of tumor-derived miRs determines the route and uptake mechanism by target stroma cells. Furthermore, damaged cells also leak miRs that fall at the interface of exosomic (encapsulated) and non-exosomic (nascent) composition. Interestingly, EVs released from tumor cells have a distinct miRome compared to parent cells, suggesting that some miRs may be transcribed only for export purposes and paracrine signaling [44,45]. Extracellular (or circulating) miRs were first detected in human blood plasma in 2008 [41,46,47]. Since then miRs have been found to circulate in a highly stable form in all other mammalian biological fluids, such as serum, milk, tears, urine, amniotic, and cerebrospinal fluid [47,48,49,50,51]. The discovery that extracellular miRs have a long half-life and are significantly altered in several diseases, including cancer, ignited great interest in using miRs as biomarkers for diagnosis and prognosis [41,47,52]. However, circulating miRs shed little light on their genesis from a particular cell and are usually derived from different cell types in a pathology; as a result, they only serve as an indicator for a pathophysiological condition. It is also possible that miRs from one cell may impact miR biogenesis in other cells.

The uptake of tumor-derived miRs also depends on the composition of exosomes. Several GTPase- and ceramide-synthesizing enzymes, such as neutral sphingomyelinase2 (nSMase2), participate in the control of intracellular formation, fusion and release of exosomes [53,54]. The membrane lipid composition and cellular lipid load of a cancer cell also determines the ‘miR pool’ of their secreted exosomes and protein-bound miRs. A notable example is the comparison of donor-derived miR uptake by macrophages during coculture with either lipid-dense breast cancer cells such as MCF-7, T4D, or lipid scarce leukemic cells such as Jurkat [38].

## 3. Macrophage Uptake of Tumor-Derived miRs—A Toll-Free Highway

**Figure 1:**
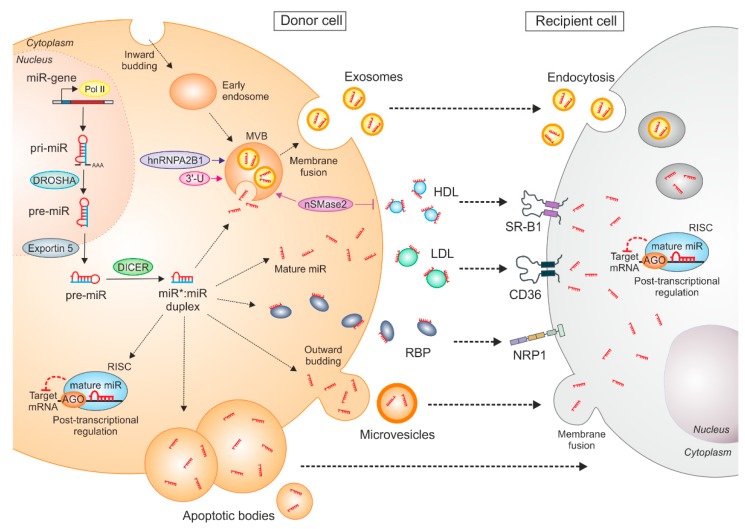
Mechanisms of miR synthesis, transfer, and intercellular communication. MiRs are transcribed by RNA polymerase II (Pol II) into primary-miRs (pri-miR), which are further processed by the DROSHA complex. The arising precursor miR (pre-miR) is exported to the cytoplasm by exportin 5 and cleaved by DICER to form a double-stranded miR:miR duplex. The mature miR is loaded onto Argonaute proteins (AGO), which together with other proteins forms the RNA-induced silencing complex (RISC). This complex binds to the 3′-untranslated region (UTR) of mRNA targets. MiRs can be exported from donor cells and taken up by recipient cells, where they repress target gene expression. Several miR carriers exist, such as extracellular vesicles (exosomes, microvesicles, apoptotic bodies), RNA-binding proteins (RBP), or high/low-density lipoproteins (HDL/LDL). MiRs can be loaded into multivesicular bodies (MVB), which are generated via early endosomal inward budding of the plasma membrane. Upon fusion of the MVBs with the plasma membrane, exosomal miRs are released into the extraluminal space. Amongst other mechanisms, the sorting of miRs into exosomes is regulated by sumoylated heterogeneous nuclear ribonucleoprotein A2/b1 (hnRNPA2B1) and miR 3′ end base modifications (3′U). Moreover, the export of miRs via exosomes is neutral sphingomyelinase 2 (nSMase2)-dependent, which, on the other hand, blocks HDL‒miR release. Exosomal miRs can be taken up by recipient cells by endocytosis or via microvesicles, which are generated by outward budding of the plasma membrane of the donor cells. Those microvesicles release their miR cargo by fusion with the plasma membrane of the recipient cells. Apoptotic bodies containing miRs are generated upon cell injury or death and are phagocytosed. HDL‒miR complexes can be taken up by recipient cells by scavenger receptor B1 (SR-B1), while the uptake of LDL‒miR is mediated by CD36. MiRs can be transferred by RBPs, such as AGO2 or nucleophosmin, which can be taken up by recipient cells via neuropilin-1 (NRP1).

While dealing with mechanisms of tumor-derived miR shuttling and uptake by macrophages, the literature primarily focused on extracellular vesicle-mediated miR transfer or the direct exchange of miRs across cells via gap junctions [55,56,57]. However, recently we discovered a novel route of tumor-derived extracellular miR transfer, which was independent of exosomes but mediated by low-density lipoproteins (LDL) [38]. In this section we provide an overview of extracellular miR release and uptake mechanisms with a focus on recent knowledge about miR transfer by lipoproteins. General modes of miR transfer are summarized in Figure 1.

### 3.1. Extracellular Vesicle-Mediated miR Transfer

Classified by their origin and biogenesis, distinct types of EVs have been described. With an average diameter of 1–5 µm, apoptotic bodies are the largest miR-carriers and are produced as byproducts of cell disassembly upon apoptosis [58,59]. Microvesicles have an average diameter of 100–1000 nm and are formed after outward budding of the plasma membrane [60]. Exosomes have a much smaller size (10–100 nm) and are primarily formed as intraluminal vesicles within multi-vesicular bodies (MVBs). They are released into the extraluminal space upon the fusion of the MVBs with the plasma membrane [60]. Besides miRs, both microvesicles and exosomes are enriched in cytoplasmic and membrane-associated proteins, lipids, sugars, and other nucleic acids [61]. Apoptotic bodies additionally include nuclear fractions and cell organelles and can be distinguished by the presence of histones [58,59,62]. Well-characterized protein markers for exosomes are annexins, tetraspanins (CD63, CD81, CD82, CD9), and heat-shock proteins (Hsp60, Hsp70, Hsp90) [60,63,64], while microvesicles contain integrins and selectins [60]. As a fourth type of EV, large oncosomes have recently been described, which are generated by membrane blebs from tumor cells [65]. However, whether they contain miRs needs to be further investigated.

At present, it is still debatable whether extracellular miRs are just released as byproducts or as a result of cell injury or death [66]. Nevertheless, increasing evidence suggests that the release and uptake of circulating miRs are tightly regulated, which is being investigated in more detail for exosomal miRs. For instance, it has been shown that only the ceramide-dependent mechanism of exosome biogenesis is involved in exosomal miR secretion [67], since the export of miR was impaired upon inhibition of nSMase2, which catalyzes ceramide biosynthesis and subsequent exosome formation [67,68]. Another possible mechanism of miR sorting into exosomes might be explained by the affinity of RNAs to raft-like membrane regions, maintaining the contact between miRs and the MVBs [69]. Moreover, several studies described differences between miR content in parental cells and their secreted exosomes [70,71,72,73,74,75,76,77], suggesting the presence of sequence-specific sorting mechanisms [74]. Indeed, Villarroya-Beltri et al. showed that sumoylated heterogenous nuclear ribonucleoprotein A2/B1 (hnRNPA2B1) controls the sorting of miRs into exosomes through binding of the specific sequence motif GGAG within miRs [77]. Additionally, non-templated nucleotide addition prior to miR polyadenylation results in the retention of miRs within the cell [72]. Nevertheless, the cellular uptake of EV-miRs can be carried out via endocytosis, phagocytosis, and micropinocytosis by recipient cells [78,79,80]. This uptake can be clatherin-dependent, as is the case for P12 cells [80]. Alternatively, endocytosis could be caveolae- and lipid-raft-dependent, as in the case of A549-P cells [81]. However, how tumor-derived miRs are sorted into exosomes and the mechanisms of EV-miR uptake by macrophages remains an open question that requires further investigation.

### 3.2. RNA-Binding Protein-Mediated miR Transfer

Besides vesicle-encapsulated miRs, a major portion of extracellular miRs have also been detected as free-floating complexes with RNA-binding proteins (RNPs), especially AGO2 [82,83] or nucleophosmin (NPM1) [66,82,84]. AGO2-bound miRs are taken up by neuropilin-1 (NRP1) and the internalized miRs remain functional, as they specifically regulate proliferation and migration of cancer cells [85]. Notably, NRP1 is highly expressed in macrophages and may be involved in AGO2-bound miR uptake. This hypothesis is further strengthened by a study that showed NRP2 expression during macrophage differentiation, which is induced by tumor cells and regulates phagocytosis in macrophages. Furthermore, NRP2 promoted efferocytosis of apoptotic tumor cell debris by TAMs and facilitated tumor growth [86]. However, it is not clear whether those miR‒protein complexes are non-selectively released after cell necrosis or whether they are exported in a regulated fashion. It has been shown that the miR content in cell media correlates with the level of cell death [83], and that the damage of certain organs leads to high amounts of tissue-specific AGO-bound miRs in the circulation [87,88,89,90,91,92]. Nevertheless, some miRs that are expressed in MCF-7 cells are absent in their cell culture supernatant, suggesting a selective export mechanism of released miRs [83]. However, whether RNP‒miR complexes are involved in tumor‒cell macrophage communication still needs to be addressed.

### 3.3. Lipoprotein-Mediated miR Transfer

Among the most critical miR carriers are the highly abundant high- and low-density lipoproteins [93]. The notion that miRs can be shuttled by lipoproteins was first established in 2011, when Vickers et al. reported that high-density lipoprotein (HDL) fractions, purified from the blood plasma of healthy human donors, contain substantial amounts of miRs [94]. They also documented that the miR profile in HDL was distinct from the miR profile in isolated exosomes. Furthermore, miR-135a*, miR-188-5p, and miR-877 were enriched in HDL particles from healthy donors, while miR-223, miR-105, and miR-106a were upregulated in HDL particles from patients with familial hypercholesterolemia [94]. In addition, they showed that HDL-mediated miR-223 transport repressed cholesterol uptake by directly targeting and downregulating the scavenger receptor BI (SR-BI) [94], adding a functional relevance to HDL‒miR complexes. Similarly, a study demonstrated that HDL-transferred miR-223 inhibited intercellular adhesion molecule 1 (ICAM1) expression in endothelial cells [95]. While the mechanism of HDL‒miR complex secretion remains unknown, it has been shown to be negatively regulated by nSMase2 [94]. Moreover, a previous study reported that HDL‒miRs are taken up by scavenger receptor BI (SR-BI) in Huh7 cells and in SR-BI-overexpressing baby hamster cells [94]. Although there are several scavenger and lipoprotein receptors expressed on macrophages [96,97], the uptake of tumor-derived HDL‒miR complexes has not been investigated.

The majority of research on lipoprotein-mediated miR transfer focused on HDL; however, in most of these studies, a substantial amount of miRs was found in the low-density lipoprotein (LDL) fractions [98,99]. For the first time, we recently demonstrated that upon coculture with MCF-7 breast cancer cells, miR-375 was elevated in TAMs [38]. This was due to the apoptosis of MCF-7 cells and the subsequent release of miR-375 as an LDL-bound non-exosomal entity, which was taken up by macrophages. MiR-375 is upregulated in breast cancer [100,101] and has already been detected in exosomes and exosome-depleted fractions of stage II and stage III breast cancer patients [102], whereas it appears as a non-exosomal LDL-bound entity in hypercholesterolemia patients [94]. With the exception of the RAW 264.7 macrophage cell line [103], TAMs and macrophages do not express miR-375 in vitro. However, they acquire it during coculture or upon their exposure to apoptotic cell-conditioned media of breast cancer cells. Treatment of macrophages with actinomycin D, which inhibits transcription and thus de novo synthesis of miRs, failed to reduce mature miR-375 levels upon coculture. Furthermore, siRNA silencing of DICER, a key miR processing enzyme, failed to reduce miR-375 in TAMs, establishing that miR-375 is tumor cell-derived. Most interestingly, miR-375 in the media was not protected from RNase A treatment, suggesting that it is not vesicle-encapsulated, since extracellular vesicles have been shown to protect miRs from endogenous RNase digestion [104]. Exosome-free extracellular miRs are sensitive to RNase treatment [82], while lipoprotein-bound miRs are likely protected from circulating RNase in the blood [105]. Contrary to this, Tabet et al. found that human coronary artery endothelial cells, when incubated with RNase treated HDL-particles, lowered the intercellular miR-223 content compared to cells incubated with untreated HDL [95]. To understand the transport mechanism of breast cancer cell-derived miR-375, it was indispensable to identify the miR-containing carrier. Apoptotic cancer cells expose oxidized LDL-like sites [106] on their surface that can be recognized by macrophage scavenger receptors such as SRA, LOX1, and CD36 [106,107]. CD36 is widely expressed and may interact with multiple extracellular ligands, including thrombospondin-1, long-chain free fatty acids, oxLDL, advanced glycation end products, and collagens I and IV [108,109]. We observed high amounts of miR-375 in the LDL fraction of serum-containing supernatants from apoptotic MCF-7 cells, suggesting a critical serum factor for miR-375 stabilization and transport. Considering the existence of LDL‒miR complexes, we asked whether the CD36 receptor might be involved in miR-375 uptake into macrophages. Blocking CD36 on macrophages with a monoclonal antibody or employing a CD36 blocking peptide attenuated the miR-375 increase in macrophages. Finally, siRNA silencing of CD36 largely prevented tumor-derived miR-375 uptake by recipient macrophages [38]. These studies provide evidence that tumor-derived miRs are not only shuttled via exosomes, but also via lipoproteins, which can be taken up by macrophage scavenger receptors. In the end, the miR pool of TAMs is composed of endogenously produced miRs and the uptake of exogeneous, tumor-derived miRs.

Still, several open questions remain. Apoptotic tumor cells release various factors including long-chain free fatty acids and oxidized phospholipids, which may also act as carriers of miR-375 for CD36-mediated uptake, as they are known ligands of CD36. Currently, it is unclear whether the CD36 receptor might be involved in the uptake of other LDL-bound miRs and whether other LDL receptors on macrophages transfer functional miRs into cells. Moreover, CD36 has been found to take up HDL-cholesterol esters [108,110] and, thus, might also be involved in HDL‒miR internalization. Future studies are required to demonstrate the functional role of the CD36 receptor in lipoprotein‒miR communication in vivo. Interestingly, CD36 has been shown to be involved in cancer metastasis [111]. The presence of CD36^+^ metastasis-initiating cells correlated with a poor prognosis for numerous carcinomas, including breast carcinoma, and the inhibition of CD36 impaired the metastasis of breast tumor cells [111]. At present, it is unknown how internalization of the miR‒lipoprotein complex via CD36 loads miRs onto the endogenous RNA-induced silencing complexes (RISC) and whether mRNAs are targeted by canonical post-transcriptional mechanisms or whether they utilize other cellular machineries to regulate target mRNA expression. Lipoprotein-bound miRs are likely single-stranded, while AGO2 is predicted to prefer double-stranded miRs. Thus, LDL‒miRs might be differentially loaded onto AGO2-RISC or other AGO family members. Moreover, LDL‒miR delivery through CD36 may bypass endosomal transport and lysosomes that may degrade extracellular RNAs, unlike receptor-mediated endocytotic mechanisms. Thus, different modes of uptake are likely associated with distinct functional integrities of the extracellular miRs in recipient cells.

## 4. Functions of Tumor-Derived miRs in Macrophages—The Trojan Horse at Work

**Figure 2:**
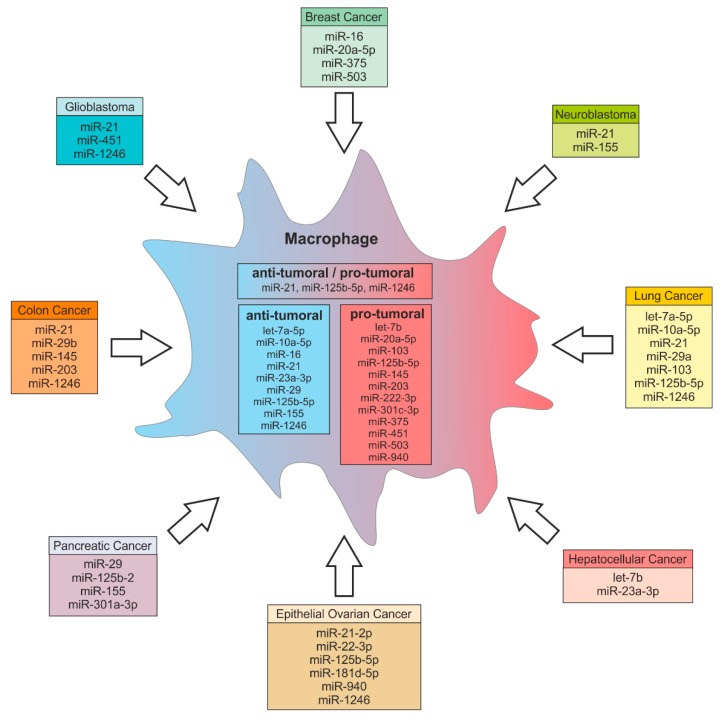
Tumor-derived miRs and their potential functions in macrophages. MiRs can be exported from various cancer types and delivered to macrophages. In macrophages those miRs regulate target gene expression, thereby inducing either anti-tumor or pro-tumor effector functions. Some miRs can have both anti-tumoral and pro-tumoral effects in recipient cells. MiRs can also target genes that indirectly regulate macrophage effector functions such as miR-375 targeting *TNS3* and *PXN* (see text for more details).

Macrophages are highly plastic cells. In response to tumor-microenvironmental cues, they undergo coordinated changes in gene expression [12,37], which may favor polarization towards a TAM phenotype. TAMs are key components in the TME and support tumor survival and growth, metastasis, angiogenesis, and immune evasion [112]. It has been shown that miRs are involved in TAM polarization. In 2012, Cay et al. demonstrated that anti-inflammatory, pro-tumor M2-like TAMs can be reprogrammed towards a pro-inflammatory M1 phenotype by miR-155 [113]. In addition, miR-19a-3p has been shown to inhibit breast cancer progression and metastasis by inducing a macrophage phenotype switch from M2 to M1 by targeting Fos-related antigen 1 (Fra-1), vascular endothelial growth factor (VEGF), and signal transducer and activator of transcription 3 (STAT3) [114]. Contrarily, miR-146a induces alternative macrophage polarization, thereby promoting 4T1 tumor growth [115]. There are other miRs expressed in TAMs that are involved in tumor progression, such as miR-142-3p [116], miR-125b [117], and miR-511-3p [118], suggesting that the deregulation of a single miR can modulate macrophage effector functions. In the following section, we will highlight the role of tumor-derived extracellular miRs in the cross-talk between different cancer types and macrophages, which mainly provokes the reprogramming of macrophages towards a pro-tumor phenotype. More recently, we identified a novel function of a breast tumor-derived miR in TAMs, which is not involved in the macrophage phenotype switch, but induced monocyte and macrophage migration and infiltration at the early stage of tumorigenesis [38]. An overview of tumor-derived miRs involved in altering the macrophage phenotype and its effector functions is depicted in Figure 2.

### 4.1. Colon Cancer

High levels of miR-203 are present in the serum of colorectal carcinoma patients and are associated with metastasis and overall poor prognosis [119,120]. A recent study showed that tumor-derived miR-203 is shuttled to monocytes, where it induces TAM differentiation in vivo to promote distant metastasis [121]. Colon cancer cells have a high expression of miR-1246, which can be delivered to macrophages via exosomes [122]. In macrophages, miR-1246 triggers anti-inflammatory immunosuppression, with an increased activity of transforming growth factor β (TGFβ) [122]. Moreover, colorectal cancer EVs containing miR-145 polarize macrophages to the M2 phenotype through downregulation of histone deacetylase 11 (HDAC11) [123].

MiR-21 is highly expressed in a variety of human tumors [124,125] and is secreted in plasma-derived exosomes from patients affected by different cancer types, including pancreatic, ovarian, lung, and colon cancer [126,127]. Elevated expression of miR-21 is associated with cell proliferation, migration, invasion, and survival and positively correlated with tumor progression [126], while its inhibition decreased tumor survival and growth [128]. Patel et al. showed that miR-21 and miR-29b are involved in a feedback loop between colorectal cancer cells and immune cells, to support the maintenance of a pro-tumorigenic inflammatory environment [129]. Immune cells secrete the pro-inflammatory cytokine IL-6, which mediates the invasiveness of tumor cells in an in vitro coculture model of colorectal cancer [130,131], thereby stimulating the secretion of miR-21 and miR-29b from tumor cells. Those miRs have been shown to bind to Toll-like receptors (TLRs) on macrophages, causing nuclear factor kappa B (NF-κB) activation and the concomitant secretion of pro-inflammatory cytokines [129].

### 4.2. Glioblastoma

Van der Vos et al. directly visualized the release of EVs from glioma cells and their uptake by microglia and monocytes/macrophages in the brain in vivo [132]. They further confirmed increased levels of miR-451/miR-21 and upregulation of c-Myc mRNA expression in recipient cells. Those cells responded with increased proliferation and shifted their cytokine profile toward immunosuppression.

Macrophages are the primary immune cell subtype in the glioma microenvironment, preferentially accumulating in hypoxic regions, where they polarize into specific phenotypes [133,134]. Qian et al. investigated the effects of glioma-derived exosomes on macrophage polarization and tumor progression [135]. In their study, hypoxic glioma-derived exosomes markedly induced macrophage M2 polarization as compared to exosomes from normoxic glioma cells. This polarization was induced by hypoxic glioma-derived miR-1246. By RNA sequencing they identified telomeric repeat binding factor 2 (TERF2IP) as an miR-1246 target in macrophages, which activated STAT3 and inhibited the NF-κB signaling pathway, thereby inducing the macrophage phenotype shift. Moreover, miR-1246 was enriched in the cerebrospinal fluid of preoperative glioblastoma patients, where it significantly decreased after tumor resection. Thus, miR-1246 might be a promising novel biomarker for glioblastoma diagnosis and a target for anti-tumor immunotherapy.

### 4.3. Lung Cancer

Tumor-derived miRs not only shape macrophage effector functions by canonical binding to their target mRNA, but also by serving as ligands of macrophage TLRs [136]. Fabbri et al. showed that non-small-cell lung cancer cells secrete EVs containing substantial amounts of miR-21 and miR-29a, which bind-s and trigger murine TLR7 and human TLR8 in macrophages and other immune cells. This process activates NF-kB pathway-mediated pro-inflammatory responses and the release of IL-6, TNFα and other pro-inflammatory cytokines, which turns the TME into a pro-metastatic niche.

Hsu et al. investigated the relationship between EVs and hypoxia upon lung cancer progression [137]. They found that EV-encapsulated miR-103a levels were higher in patients with lung cancer and closely associated with macrophage M2 polarization. Hypoxia induced the secretion of miR-103a containing EVs from lung cancer cells [138], which were transferred to macrophages and genetically inactivated tumor suppressor phosphatase and tensin homolog (PTEN). Consequently, protein kinase B (AKT) and STAT3 activation was increased, as well as the expression of angiogenic VEGF-A and angiopoietin-1 from M2 macrophages. Thus, miR-103a signaling induced a feedback to further enhance cancer progression and angiogenesis. On the other hand, lung adenocarcinoma cell-derived exosomal miR let-7a-5p, miR-10a-5p, miR-1246, and miR-125b-5p promoted macrophage reprogramming towards a dominating pro-inflammatory, anti-tumor M1 phenotype [139].

### 4.4. Hepatocellular Carcinoma

A recent study by Liu et al. found that endoplasmic reticulum stress caused liver cancer cells to release exosomal miR-23a-3p, which was delivered to macrophages [140]. In macrophages, miR-23a-3p inhibited PTEN and subsequently activated AKT, which increased the expression of programmed cell death ligand 1 (PD-L1) and inflammatory cytokines. Treatment of macrophages with those miR-containing exosomes upregulated PD-L1 expression, both in vitro and in vivo. Furthermore, an in vitro coculture of those exosome-treated macrophages with T cells, reduced the number of CD8^+^ T cells and the production of interleukin-2, while T cell apoptosis was increased [140]. These findings provide another mechanism of how tumor cells escape anti-tumor immunity mediated by miRs.

Li et al. also investigated the role of TLR signaling pathways in the regulation of tumor inflammation [141]. TLR4 signaling of H22 hepatoma cells was transduced through myeloid differentiation primary response 88 (MyD88) to the actin cytoskeleton, which caused the release of miR let-7b containing membrane-derived vesicles by the tumor cells. These microvesicles were taken up by macrophages, where miR let-7b targeted the pro-inflammatory cytokine IL-6 to attenuate tumor inflammation.

### 4.5. Breast Cancer

Jang et al. found that treatment of the murine 4T1 breast cancer cells with the anti-tumor drug epigallocatechin gallate (EGCG) decreased the expression of macrophage colony-stimulating factor 1 (CSF-1) and CC-chemokine ligand 2 (CCL2), which inhibits TAM infiltration and migration [142]. Additionally, EGCG upregulated miR-16 in 4T1 cells, which was transferred to TAMs via exosomes. Those exosomes suppressed IKKα, caused I-κB accumulation and the subsequent repolarization of M2 to M1 macrophages. Effects on chemokine expression and the NF-κB pathway were restored when miR-16 was inhibited in EGCG-treated cancer cells. MiRs have also been linked to breast cancer metastasis. In microglia, exosomal miR-503 promoted polarization from tumor-suppressive M1 to the pro-tumor M2 phenotype, which contributed to brain metastasis in breast cancer [143]. Guo et al. investigated mechanisms of breast cancer invasion to the bone marrow [144]. They noticed that, in breast tumor tissues as well as in exosomes from MDA-MB-231 cells, miR-20a-5p was highly elevated. In MDA-MB-231 cells, miR-20a-5p promoted migration and invasion. Furthermore, exosomal miR-20a-5p was delivered to bone marrow macrophages and stimulated osteoclastogenesis by targeting SRC kinase-signaling inhibitor 1 (SRCIN1).

In our recent study, we showed that apoptotic breast cancer cell-derived miR-375 is shuttled to macrophages via LDL and induced macrophage migration and infiltration [38]. In breast cancer, TAMs are the predominant immune cell subtype and most of them originate from blood-derived monocytes [145,146]. Currently, it is unclear how the tumor microenvironment achieves this massive influx of monocytes and macrophages and how it initiates genetic reprogramming of TAMs. While tumor-derived miRs that alter the macrophage phenotype have been reported in this review and elsewhere [123], our study provides evidence that miR-375 provokes monocyte/macrophage migration and infiltration without affecting cell polarization [38]. To identify miR-375 targets in macrophages, we overexpressed miR-375 using a synthetic miR-mimic and subsequently performed AGO-RIP-Seq. On the basis of the results we obtained from sequencing, we identified paxillin (*PXN*) and tensin 3 (*TNS3*) as targets of miR-375 in macrophages, which was verified in vitro and in vivo. Paxillin and tensin 3 mRNA and protein levels were reduced in macrophages upon coculture with breast cancer cells, as well as after treatment with miR-375 containing supernatants from apoptotic MCF-7 cells. Paxillin is a focal adhesion adapter protein that, depending on its subcellular localization, can positively or negatively regulate cell migration [147,148,149]. Similarly, tensin 3 acts as a link between the extracellular matrix and the cytoskeleton and functionally contributes to the switch between adhesive and non-adhesive states in cancer cells, including breast cancer [150,151]. In tissue microarrays of mammary carcinoma patients, miR-375 was elevated in breast cancer tissue as compared to normal breast tissue, and positively correlated with the number of infiltrated macrophages. These findings highlight the important role of tumor-derived miR-375 in the early stages of tumorigenesis, specifically in the recruitment of circulating monocytes to sites of tumor progression, which makes miR-375 a promising new therapeutic target at the onset of breast cancer.

### 4.6. Epithelial Ovarian Cancer

Tumor-derived extracellular miRs also take part in macrophage polarization in epithelial ovarian cancer (EOC). MiR-222-3p is shuttled to macrophages, where it downregulates the suppressor of cytokine signaling 3 (SOCS3) and subsequently favored STAT3 phosphorylation [152]. This process caused macrophage polarization towards a TAM-like phenotype, which promoted the progression of EOC. Furthermore, exosome-derived miR-940 was released by hypoxic EOC and induced TAM polarization [153]. Another study showed that, under hypoxic conditions, exosomal miR-21-2p, miR-125b-5p, and miR-181d-5p were delivered to PMA-treated U937 cells and induced polarization towards TAMs in vitro and in vivo [154]. This process was facilitated by the downregulation of SOCS4/SOCS5, increased STAT3 phosphorylation, and subsequent HIF-1α/HIF-2α stabilization. Those TAMs promoted EOC cell proliferation and migration in a feedback loop.

MiRs are also involved in tumor cell resistance to chemotherapy. Kanlikilicer et al. overexpressed caveolin 1 (CAV1), a direct target of miR-1246, which significantly sensitized EOC cells to paclitaxel [155]. This was confirmed by decreased multidrug-resistance protein (MDR1) protein levels. Furthermore, anti-miR-1246 treatment in combination with paclitaxel significantly reduced tumor growth in vitro as well as the tumor burden in an orthotopic SKOV-ip1 EOC mouse model. Upon coculture with EOC cells, miR-1246 was highly elevated in M2-type macrophages, but not in M0-type macrophages. Conclusively, miR-1246 may act both autonomously by inhibition of CAV1, and also as an extracellular mediator that promotes tumor progression via M2-type oncogenic macrophages.

### 4.7. Pancreatic Cancer

Pancreatic cancer cells (PC) have also been shown to reprogram pro-inflammatory macrophages to M2-like pro-tumorigenic macrophages via exosomal miRs. Su et al. demonstrated that exosomes from cells transfected with miR-155 and miR-125b-2 expressing plasmids are enriched in these miR [156]. The uptake of those miR containing exosomes by macrophages resulted in their reprogramming towards the M1 phenotype. In another study, exosomal miR-301a-3p derived from hypoxic pancreatic cancer cells activated the PTEN/phosphoinositide-3-phosphate gamma pathway in macrophages, thereby triggered macrophage M2 polarization and increased the metastatic activity of PC [157]. The exosomal transfer of miR to innate immune cells is not limited to cancer cells as miR donors. Pancreatic beta-cells transferred exosomal miR-29 to macrophages to dose-dependently increase TNFα secretion by recipient cells [158].

### 4.8. Neuroblastoma

Challagundla et al. showed that miRs can be involved in a feedback loop generating chemotherapy resistance in neuroblastoma [159]. Tumor-derived exosomes delivered miR-21 to TAMs, which activated TLR8 and upregulated the expression of the oncomiR miR-155. MiR-155 is transcriptionally regulated by the NF-κB pathway and overexpressed in multiple types of cancer. Subsequently, TAMs release exosomes enriched in miR-155, which is taken up by tumor cells, where it targets telomeric repeat binding factor 1 mRNA. The resulting activation of telomerase activity ultimately accounts for resistance of neuroblastoma to cisplatin treatment.

### 4.9. Other Cancers

Park et al. investigated the role of oxygen depletion on tumor exosome cargo and release [160]. They found that, upon hypoxia, miR let-7a, a known epigenetic tumor suppressor, was decreased in murine B16-F0 tumor cells [161], but was increased >20-fold in exosomes. In turn, the exosomal transfer of miR let-7a enhanced OXPHOS activity and M2-like polarization of bone marrow macrophages via downregulation of the insulin AKT-mTOR pathway. While miR let-7a was enriched in hypoxic exosomes, target gene expression was significant in both normoxic and hypoxic exosome-treated cells, suggesting that further characterization of miR let-7a targets is required. Microvesicle delivery of miR-150 from THP-1 cells promoted the enhanced secretion of VEGF from TAMs, thereby stimulating tumorigenesis [162]. Hsieh et al. investigated the role of epithelial‒mesenchymal transition (EMT) in remodeling the tumor microenvironment [163]. They demonstrated that the EMT transcriptional factor SNAIL directly activated the transcription of *MIR21*. Subsequently, mature miR-21 was delivered to CD14^+^ human monocytes via exosomes and suppressed the expression of M1 markers, while increasing M2 markers. SNAIL-induced M2 polarization was attenuated by knocking down miR-21 in *SNAIL*-expressing human head and neck cancer cells.

**Figure 3:**
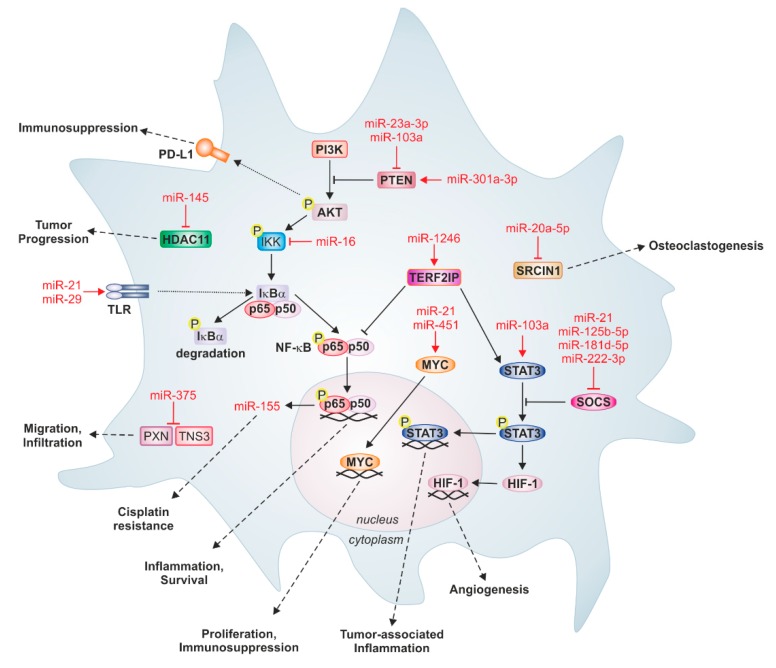
Prominent tumor-derived miRs and their targets in macrophages. Tumor-derived miRs can target several important signaling pathways in recipient macrophages, which are associated with inflammation and survival, proliferation, immunosuppression, tumor-associated inflammation, and angiogenesis. For instance, binding of miR-21 and miR-29 to Toll-like receptors (TLR) activates nuclear factor kappa B (NF-κB) signaling and increases the expression of pro-inflammatory cytokines. Likewise, miR-103a genetically inactivates tumor suppressor phosphatase and tensin homolog (PTEN), thereby increasing protein kinase B (AKT) and signal transducer and activator of transcription 3 (STAT3) activation, causing cancer progression and angiogenesis. Some miRs induce monocyte differentiation. MiR-20a-5p from breast cancer cells targets SRC kinase-signaling inhibitor 1 (SRCIN1) in bone marrow macrophages, which stimulates osteoclastogenesis. Moreover, apoptotic breast cancer-derived miR-375 downregulates the migration inhibitory proteins paxillin (PXN) and tensin 3 (TNS3) in monocytes and macrophages, thereby provoking infiltration into primary tumors. Additionally, miRs can also be involved in feedback loops between tumor cells and macrophages; tumor-derived miR-21 binds TLR8 on TAMs, which activates NF-κB signaling and the subsequent expression of miR-155, which can be secreted by TAMs to induce cisplatin resistance in neuroblastoma cells. HDAC11: histone deacetylase 11; HIF-1: hypoxia-inducible factor 1; IKK: IκBα‒kinase complex; MYC: Myc proto-oncogene protein; PI3K: phosphoinositid-3-kinase; PD-L1: Programmed cell death 1 ligand 1; SOCS: suppressor of cytokine signaling; TERF2IP: telomeric repeat binding factor.

Taken together, tumor-derived exosomic and non-exosomic miRs represent a ‘virus-like’ gene regulating tool, which is efficiently used by tumor cells to affect immune surveillance and to induce immune suppression by hijacking immune sentinel such as macrophages. The broad specificity of miRs towards their targets can also be exploited by tumor cells. For instance, tumor-derived miR-375 induced monocyte/macrophage migration towards the TME by downregulating negative regulators of cell migration, viz. *TNS3* and *PXN.* MiR-375 also potentiated these migratory effects on monocytes/macrophages by inducing macrophage chemoattractant CCL2 production by tumor cells in the same setting [38]. Furthermore, as discussed in this section, tumors have evolved to evade anti-tumor immunity by targeting various signaling pathways in macrophages, which are crucial for their anti-tumor functions, via tumor-derived miRs. This phenomenon also provides an opportunity to exploit this gateway for anti-tumor therapy. A brief summary of tumor-derived miR targets in macrophages is presented in Figure 3.

## 5. Macrophage-Derived miRs—An Arm of a Sentinel

Macrophages are not exclusive targets for miR tumor Trojan horses; rather, they are sentinels of the immune system fighting against tumors, and macrophage-derived miRs act as one arm of these innate immune functions. There are several examples in the literature that consolidate the function of these cells as mediators of tumor immunosurveillance [164]. A notable example of this phenomenon is the study by Aucher et al., investigating the role of exosomal miR-223 in cancer suppression. It appears that exosomes containing miR-223 and miR-142 were released by monocyte-derived macrophages and transported to cocultivated hepatocellular carcinoma cells in a manner that required intercellular contact and gap junctions. Such a transfer inhibited the proliferation of cancer cells [165]. The tumor suppressing functions of miR-142-3p delivered in microvesicles from TAMs to hepatocellular tumor cells were demonstrated in a mouse model. Animals bearing tumors were treated with anti-tumor propofol. The downregulation of miR-142-3p in TAMs using its inhibitor reversed the effect of propofol on hepatocellular carcinoma cells [166].

In addition to the transfer of endogenous macrophage-derived miRs in the TME, some studies aiming for therapeutic options documented miRs that are either induced by the treatment of macrophages [167] or using macrophage-derived vesicles as drug delivery systems [168,169]. Hu et al. treated macrophages with the tumor necrosis factor (TNF)-like weak inducer of apoptosis (TWEAK), which has been reported to potentially mediate an anti-tumor effect for tumor-infiltrating macrophages [170,171]. Apparently, exosomes derived from human THP-1 monocyte-derived macrophages that were stimulated with TWEAK inhibited metastasis and invasiveness of EOC tumors in vitro and in vivo. Inhibition was provoked by a TWEAK-induced enrichment of miR-7 in macrophages and the miR-7 transfer to the EOC cells, where it downregulated the EGFR/AKT/ERK1/2 signaling pathways [167]. Wang et al. showed that an exogenous miR-21 inhibitor could be shuttled into BGC-823 gastric cancer cells via macrophage-derived exosomes, which provided evidence that macrophage-derived exosomes could function as carriers for therapeutic miR agents [168].

Macrophage-derived miRs play an important role in the communication between macrophages and surrounding cells—not only in the cancer context, but also during macrophage development. An miR that has been shown to be highly expressed in macrophage-derived microvesicles is miR-223. The transfer of miR-223 can stimulate myeloid proliferation [172] and induce macrophage differentiation in recipient monocytes [173]. This dual role is supported by different studies, either showing a pro- or anti-tumor effect of macrophage-derived miRs in recipient cells. Furthermore, in the EOC context, miR-223 is transferred from hypoxic macrophages to cocultured EOC cells via exosomes. This exosome transfer enhanced drug resistance via miR-223-mediated regulation of the PTEN-PI3K/AKT pathway both in vivo and in vitro [174].

In essence, tumor cells can use miRs to hijack stroma for pro-tumoral functions. Similarly, inherent anti-tumoral macrophages use miRs to exert their anti-tumor activity. This double-edged sword nature of miRs poses a challenge for their use as RNA therapeutics.

## 6. Macrophage-Derived miRs—Agent Gone Rogue

TAMs have the ability to promote tumor invasion and metastasis. Apart from other soluble factors, macrophages also perform pro-tumoral functions by secreting oncomiRs, which regulate gene expression in recipient tumor cells. A notable example of this aspect of TAM biology is the secretion of EVs containing high levels of miR-233, which can be transferred to breast cancer cells to promote their invasiveness by regulating the myocyte enhancer factor 2c (Mef2c)-β-catenin signaling pathway. Reducing Mef2c elicits nuclear accumulation of β-catenin to promote the invasiveness of breast cancer cells [175]. However, the effect can be diminished by attenuating miR-223 expression via the antisense oligonucleotide. In pancreatic ductal adenocarcinoma (PDAC) TAM recruitment is associated with metastasis. Yin et al. ascribed this to the exosome-mediated transfer of miR-501-3p from M2 macrophages to PDAC cells. In a mouse model, they showed inhibition of the tumor suppressor gene TGFBR3 by miR-501-3p and subsequent activation of the TGF-β signaling pathway [176]. Another study, employing the genetic mouse model of PDAC, showed that the transfer of macrophage-derived miR-365 via exosomes impaired gemcitabine activation, thereby conferring therapy resistance in vitro and in vivo. As the underlying mechanism, the authors proposed upregulation of the triphospho-nucleotide pool in cancer cells and induction of the enzyme cytidine deaminase [177]. Zheng et al. showed that TAM-like M2 macrophage-derived miR-21 is transferred to MFC and MGC-803 gastric cancer cells via exosomes. In cancer cells, miR-21 downregulates PTEN and thereby activates the PI3K/AKT pathway, giving rise to drug resistance against DDP treatment in vitro and in vivo. Additionally, treatment of the cancer cells with exosomes or miR-21 overexpression protected cells from chemotherapy-induced apoptosis via increased Bcl-2 expression [178]. Macrophage oncomiRs also have context-dependent effects. Korabecna et al. analyzed target genes of two macrophage-derived miRs, miR-223 and miR-142-3p, which were experimentally proven to be functional in recipient cancer cells. The two miRs targeted 684 genes, amongst them tumor suppressors like TP53 or adenomatous polyposis coli. This points to a potential role of macrophage-derived miRs in cancer cell proliferation. Furthermore, it was reported that 34 targeted genes are involved in ‘pathways in cancer’ using DAVID (database of annotation, visualization and integration discovery) analysis. As this group of genes comprises oncogenes as well as tumor suppressor genes, the authors propose a potential dual role of TAMs, depending on the actual state of the TME [179]. MiRs that are involved in tumor‒stroma cross-communication are listed in Table 1.

## 7. Conclusions and Future Perspectives

Apart from being essential regulators of physiological functions, miRs are evidently used in pathophysiological exploitations by cancers. A salient feature of miRs is to aid in tumor cell‒stroma crosstalk. This crosstalk takes place by different modes of miR transfer between tumor cells and stromal components, such as macrophages, or vice versa, as depicted in Figure 1. Depending upon the molecular composition of miR‒carrier complexes, only a specific transfer route allows the miR to enter the target cell and regulate target genes, as discussed in Section 3. Tumor cells secrete various miR species (Figure 2) that target various pathways, which are involved in macrophage effector functions (Figure 3). This elegantly choreographed exploitation of immunity by tumor cells provides us with ample opportunity for therapeutic intervention.

As an RNA species, miRs are prime candidates for use in RNA therapeutics. Since several miRs are reported to be dysregulated in cancer, both loss-of-function and gain-of-function approaches have been used as therapeutic options. Several pre-clinical studies and registered clinical trials adopted these approaches. Intratumoral injections of miR drugs directly into the pathogenic site can enhance target specificity, efficacy, and minimize side effects [180,181]. Phase II clinical trials of the antagomir drug MRG-106, a locked nucleic acid (LNA)-modified antisense oligonucleotide, to inhibit the function of miR-155 are underway for cutaneous T-cell lymphoma (NCT03713320). Similarly, MesomiR-1, the tumor suppressor miR-16 mimic, was used in nonliving bacterial minicells to target EGFR-expressing non-small-cell lung cancer cells with an anti-EGFR bispecific antibody (NCT02369198) [182].

This review may serve as a primer to understand the intricacy of miR-mediated cross-communication in the TME and may pave the way to designing therapeutic interventions. To achieve this objective, it is important that we understand how cells communicate through miRs and which critical parameters should be taken into consideration. Like in a basic telecommunication system, the quality and success of miR-mediated intracellular communication also depends on basic elements: (a) a transmitter cell that relays the information as a ‘signal’, (b) a transmission channel that carries the signal, such as exosome or LDL-bound miR and (c) a receiver cell that gathers the signal from the channel and converts it back into usable information for the recipient. In this communication scheme, all three components have equal importance and a successful message relay and ‘therapeutic interception’ depends on how well we understand these elements. The last decade has been dedicated to deciphering and exploiting a canonical feature of miRs, i.e., intercellular gene regulation. Non-canonical functions of miRs, especially in the context of pathophysiological situations such as cancer, are now in the limelight. It is incumbent for successful RNA therapeutics, especially receptor-mediated delivery of therapeutic RNA, that miR‒carrier complex composition and the mode of delivery are taken into consideration. In conjunction with the emphasis on deciphering the transport mechanism, the development of robust and reliable techniques is required to track the origin and fate of miRs in vivo.

Based on the recent findings on various modes of miR delivery to target cells and several distinct package compositions of miR‒carrier complexes, the immediate focus might be on (a) the identification of particular miR preferential target(s), (b) how miRs escape extra- and intra-cellular degradation, and (c) the molecular mechanism of their target cell entry to promote gene silencing. Addressing these aspects will be crucial in designing effective RNA therapeutics. For exosomic miRs, some form of selectivity is provided by the proteins located on the exosome membrane that interact with the host cell. However, target cell selectivity of LDL-bound or AGO2-bound miRs is still an open question because receptors for these complexes such as CD36 and NRP-1 and NLR-2 are widely expressed on stroma cells. Furthermore, it would be interesting to understand how LDL- or AGO2-bound miRs, once taken up by phagocytes, are integrated into the RISC complex for gene silencing and how phagocytes are programmed to funnel these bound miRs for effective sorting to the RISC and not to lysosomes for degradation. These open questions also point to the fact that an extra layer of inter- and intra-cellular communication exists that may define the fate of receptor-mediated miR uptake in TAMs, which could be crucial for tumor-specific therapeutic RNA targeting.

## Figures and Tables

**Table 1:** MiR-mediated cross-communication between tumor cells and stroma.

miR	Donor cells	Acceptor cells	Function	Ref.
miR-203	Colon cancer cells	Monocytes	Induction of TAM differentiation	[121]
miR-1246	Colon cancer cells	Macrophages	Immunosuppression	[122]
	Hypoxic glioma cells	Macrophages	M2 macrophage polarization	[135]
	EOC cells	M2 macrophages	M2 macrophage polarization	[155]
miR-145	Colorectal cancer cells	Macrophages	M2 macrophage polarization	[123]
miR-103a	Hypoxic lung cancer cells	Macrophages	Cancer progression, angiogenesis	[138]
miR let-7a-5p, miR-10a-5p, miR-1246, miR-125b-5p	Lung adenocarcinoma cells	Macrophages	M1 macrophage reprogramming	[139]
miR let-7a	Hypoxic melanoma B16-F0 cells	Bone marrow macrophages	Enhanced oxidative phosphorylation activity, M2 macrophage polarization	[161]
miR let-7b	Hepato-carcinoma cells	Macrophages	Attenuation of tumor inflammation	[141]
miR-23a-3p	Liver cancer cells	Macrophages	Immune evasion	[140]
miR-16	EGCG-treated 4T1 breast cancer cells	TAMs	Repolarization to M1 macrophages	[142]
miR-503	Breast cancer cells	Microglia	M2 macrophage polarization	[143]
miR-20a-5p	MDA-MB-231 cells	Bone marrow macrophages	Stimulation of osteoclastogenesis	[144]
miR-375	Breast cancer cells	Macrophages	Macrophage migration/infiltration	[38]
miR-222-3p	EOC cells	Macrophages	TAM polarization	[152]
miR-940	Hypoxic EOC cells	Macrophages	TAM polarization	[153]
miR-21	Head and neck cancer cells	CD14^+^ human monocytes	M2 macrophage polarization	[163]
	Neuroblastoma cells	TAMs	Activation of TLR8, upregulation of miR-155	[159]
miR-21-3p, miR-125b-5p, miR-181d-5p	EOC cells	PMA-treated U937 cells	TAM polarization	[154]
miR-21, miR-29a	NSCLC cells	Macrophages	TLR activation, activation of NF-kB signaling and pro-inflammatory cytokine secretion	[136]
miR-21, miR-29b	Colorectal cancer cells	Macrophages	TLR activation, activation of NF-kB signaling and pro-inflammatory cytokine secretion	[129]
miR-21, miR-451	Glioma cells	Microglia, monocytes/macrophages	Increased proliferation, immunosuppression	[132]
miR-301a-3p	Hypoxic pancreatic cancer cells	Macrophages	Macrophage M2 polarization	[157]
miR-150	THP-1 cells	TAMs	Promotion of tumorigenesis	[162]
miR-29	Pancreatic beta cells	Macrophages	Increased TNFα secretion	[158]
exogenous miR-155, miR-125b-2	Transfected pancreatic cancer cells	Macrophages	Repolarization to M1 macrophages	[156]
miR-155	TAMs	Neuroblastoma cells	Cisplatin resistance	[159]
miR-223	IL-4 activated macrophages	Breast cancer cells	Increased invasiveness	[175]
	Hypoxic macrophages	EOC	Enhanced drug resistance	[174]
miR-223, miR-142-3p	Macrophages	Hepato-carcinoma cells	Inhibited cancer cell proliferation	[165]
miR-142-3p	TAMs	Hepato-carcinoma cells	Conveys propofol effect	[166]
miR-21	TAM-like M2 macrophage	Gastric cancer cells	Drug resistance, reduced apoptosis	[178]
miR-501-3p	M2 macrophages	PDAC cells	Metastasis	[176]
miR-365	Macrophages	PDAC cells	Gemcitabine resistance	[177]
miR-7	TWEAK-stimulated macrophages	EOC cells	Inhibits metastasis and invasiveness	[167]
